# Cynaropicrin inhibits pancreatic cancer cell viability and disrupts cellular redox homeostasis

**DOI:** 10.17912/micropub.biology.002097

**Published:** 2026-04-06

**Authors:** Heather R Leek, Abby L Oberhelman, Sam R Rew, Olivia M Janss, Cole D Davidson

**Affiliations:** 1 Biology, Wartburg College, Waverly, IA, US

## Abstract

Pancreatic cancer accounts for approximately 8% of all cancer-related deaths. The compound cynaropicrin (CNP) has been investigated recently in several cancer models and has been shown to decrease cell viability. We used sulforhodamine B and trypan blue assays to measure cell viability following CNP treatment in PANC-1 cells. CNP reduced cell viability in a concentration-dependent manner (IC _50_ = 5.29 µM) and significantly decreased free thiol levels. Finally, *in silico* docking demonstrated the potential for CNP to bind the DNA-binding domain of NF-κB. These data support further investigation of CNP as a potential therapeutic candidate in pancreatic cancer.

**Figure 1. Cynaropicrin decreases proliferation and free thiol levels in PANC-1 cells:** **(A)** Cynaropicrin inhibits pancreatic cancer cell viability determined using an SRB assay. **(B)** IC _50_ determination of CNP from the SRB assay (5.29 μM). **(C)** Microscope images of SRB-stained PANC-1 cells treated with DMSO. 40X total magnification. Scale bar = 500 μm. **(D)** Microscope images of SRB-stained PANC-1 cells treated with 10 μM CNP. 40X total magnification. Scale bar = 500 μm. &nbsp; **(E)** Total alive cells counted via trypan blue assay following 72 hours of treatment with DMSO or 10 μM CNP. **(F)** Percentage of counted dead cells in the trypan blue assay following 72 hours of treatment with DMSO or 10 μM CNP. **(G)** Relative concentration of free thiols following 24 hours of treatment with DMSO, 5.29 μM CNP, or 750 μM H _2_ O _2_ . **(H)** Image of simulated CNP docking with NF-κB. Significance between multiple groups was determined by one-way ANOVA followed by Dunnett’s multiple comparison tests where * represents p < 0.001 and # represents p < 0.0001. CNP concentrations that are not denoted were not significantly different from the control group (p ≥ 0.05). Significance between two groups was determined by a two-tailed, unpaired Student's t-test, and *** represents p < 0.001. Error bars represent standard deviation.

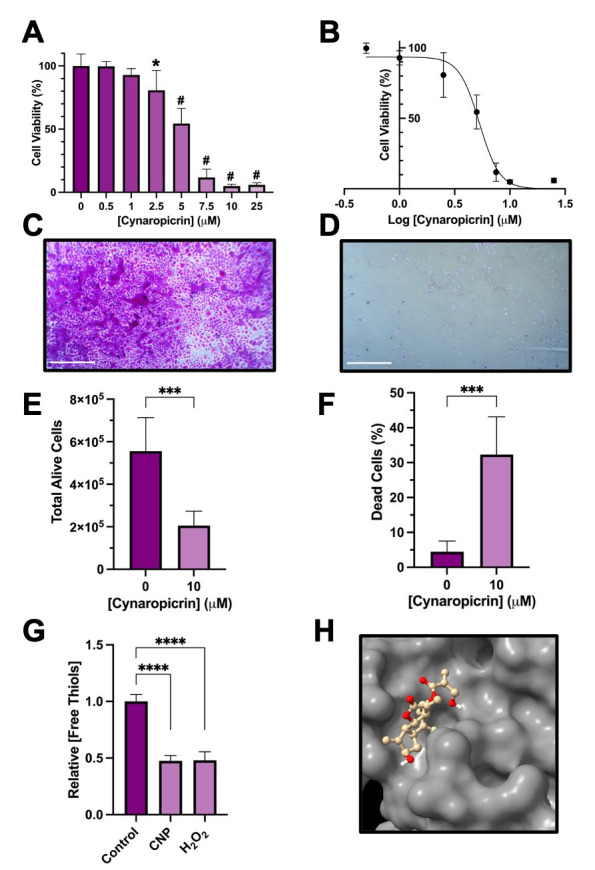



## Description

The cause of pancreatic cancer is highly variable amongst patient cases; however, common hypotheses suggest that accumulation of oncogenic mutations leads to uncontrolled proliferation. Pancreatic cancer is often undetected until later stages due to its location, lack of early-stage symptoms, and limited screening options. Current standard-of-care chemotherapy includes gemcitabine and FOLFIRINOX, but the 5-year survival rate for patients is only 23.7% (Li et al., 2022). This poor prognosis has driven current research to focus on novel treatments. Cynaropicrin (CNP), a sesquiterpene lactone derived from *Cynara scolymus* leaves, exhibits antiproliferative effects in multiple cancer models such as colorectal, breast, brain, and hematologic malignancies (Zheng et al., 2021; Amaral et al., 2026; Yang et al., 2022a; Rotondo et al., 2022; Boulos et al., 2023). Our study investigated the potential effects of CNP in pancreatic cancer cells. We hypothesized that CNP would reduce pancreatic cancer cell viability.

The sulforhodamine B (SRB) assay was performed to determine the viability of the PANC-1 cell line after treatment with DMSO alone or CNP concentrations ranging 0.5-25 µM for 72 hours. CNP decreased viability of cells in a concentration-dependent manner (A). CNP concentrations of 0.5 and 1 µM did not have significant impact on cell viability. However, a decrease in viability could be detected starting at 2.5 µM. This assay was used to find the half maximal inhibitory concentration (IC _50_ ), which was determined to be 5.29 μM with a 95% confidence interval of 4.97-5.57 μM (B). Phase-contrast microscopy visualized CNP’s ability to reduce cell growth compared to the control cells (C, D).

One limitation of the SRB assay is that it stains apoptotic cells. Therefore, we implemented the trypan blue assay to complement the SRB results (E). Trypan blue is excluded from cells with intact cell membranes, so it can identify late-apoptotic cells. Cells that were stained with trypan blue following 72 hours of 10 µM CNP treatment contained 32.3% dead cells compared to 4.49% dead cells seen in the control population (F).

Many cytotoxic compounds such as CNP have been shown to reduce cell viability by increasing the levels of reactive oxygen species (ROS) (Rotondo et al., 2022). There is an inverse relationship between ROS levels and free thiols, which are most often found in cysteine protein residues. Therefore, we used Ellman’s reagent to measure free thiol groups as an indirect measure of oxidative stress in CNP-treated cells. Compared to DMSO-treated cells, the relative concentration of free thiols was less than half in the CNP-treated cells and cells treated with a positive control, H _2_ O _2_ (G). Since disruption of the NF-κB signaling pathway can lead to ROS accumulation, we performed *in silico* docking analysis to determine if CNP could theoretically bind to and inhibit NF-κB. Although CNP has been reported to inhibit NF-κB, a specific pharmacological mechanism has not yet been proposed to our knowledge (Tanaka et al., 2013). Structural analysis using CB-Dock2 calculated a Vina score of -6.7, suggesting a potential interaction between CNP and the DNA-binding domain of NF-κB (Jain and Sudandiradoss 2022). CNP is predicted to bind NF-κB residues that are essential for NF-κB binding to DNA: Y59, V60, A61, A110, H111, S112, L113, V114, E119, D120, G121, I122, L142, H143, V144, T145, K148, E151, T152, A155, and R156 (H). NF-κB is a particularly relevant target in pancreatic cancer, where NF-κB signaling induces expression of ROS scavenger genes in response to high levels of oxidative stress (Kabacaoglu et al., 2019). Notably, our docking analysis predicted that CNP interacts with residues within the NF-κB DNA-binding region, suggesting that CNP could interfere with NF-κB transcriptional activity. Such inhibition could potentially impair NF-κB-mediated antioxidant responses, thereby contributing to ROS accumulation and decreased levels of free thiols. This interpretation is consistent with previous work in PANC-1 cells showing that NF-κB inhibition resulted in apoptosis and ROS accumulation (Lau et al., 2010).

Taken together, our results demonstrated that CNP is toxic to the pancreatic cancer cell line, PANC-1. CNP reduced cell viability, increased cell death, decreased free thiol levels, and was predicted to bind NF-κB *in silico* . The IC _50_ value was comparable to other studies in colorectal cancer (3.892-8.885 μM) (Zheng et al., 2021), breast cancer (8-10 μM) (Amaral et al., 2026), neuroblastoma (5.738-9.731 μM) (Yang et al., 2022a), glioblastoma (3.1 μM) (Rotondo et al., 2022), multiple myeloma (1.8-3.2 μM), and leukemia (2.6-2.9 μM) (Boulos et al., 2023). This work should be validated in other pancreatic cancer cell lines to increase generalizability, and due to its low IC _50_ value, CNP has strong potential for further investigation in pancreatic cancer xenograft models.

## Methods

Cell Culture

PANC-1 cells (American Type Culture Collection, Manassas VA, USA) were cultured in Eagle’s Minimum Essential Media (Corning, Corning NY, USA) with 10% FBS, penicillin (100 IU/L) (ThermoFisher Scientific, Waltham MA, USA), and streptomycin (100 µg/mL) (Corning). Cells were incubated for at least two days at 37 °C, 100% humidity, and 5% CO _2_ before experimentation. When cells reached ~70% confluence, media was aspirated, and cells were washed with warm PBS (ThermoFisher) and two mL of 0.25% trypsin (ThermoFisher) were added for five minutes at 37 °C to lift cells from the plate. The trypsin was neutralized with eight mL of media and collected cells were vortexed briefly and cell counts were obtained with a hemocytometer. All experiments were conducted using PANC-1 cells within one to five passages after thawing.

Sulforhodamine B Cell Viability Assay

Cells (2.5 X 10 ^3^ ) were seeded in 96 well plates (Genesee Scientific, El Cajon CA, USA) with 100 μL media and left to adhere overnight in the incubator. Then, 100 µL of CNP (MedChemExpress, Monmouth Junction NJ, USA) solution prepared at 2X the target final concentration were added to each well containing 100 µL of culture medium (1:1 dilution), resulting in the indicated final treatment concentrations. Cells were then incubated for 72 hours. The control media and each concentration of CNP contained the same percent of DMSO: 0.05% (DMSO: UFC Biotechnology, Buffalo NY, USA). After incubation, 100 µL of 10% w/v cold trichloroacetic acid (ThermoFisher) were added to each well and the plate was incubated for one hour at 4 ºC to fix the cells. The cell liquid was forcefully poured out, and the wells were washed three times with DI H _2_ O and stained with 100 µL of 0.057% w/v sulforhodamine B (SRB, Sigma-Aldrich) for 30 minutes at room temperature. Each well was then washed with 300 µL of 1% v/v acetic acid (ThermoFisher) and the remaining SRB was solubilized with 200 µL of 10 mM tris buffer, pH 10.5 (ThermoFisher). Absorbance was measured at 564 nm with a SpectraMax 190 Microplate Reader (Molecular Devices, San Jose, CA, USA) and normalized to the DMSO control. Cells were also imaged at 40X magnification with an Olympus BX41 phase contrast microscope (Hachioji, Tokyo, Japan) with an AmScope HD202-MW camera (Irvine, CA, USA).

Trypan Blue Assay

The trypan blue assay was conducted as described previously (Ashing et al., 2024). Briefly, cells (1 X 10 ^5^ ) were seeded in six-well plates (Advangene Consumables, Lake Bluff, IL, USA) with two mL of media per well and incubated at 37 °C overnight to adhere. The media were then removed, and cells were treated with DMSO or 10 µM CNP. After 72 hours, the media were aspirated and cells were washed with PBS, incubated for five minutes with 200 µL of 0.25% trypsin at 37 °C, collected with 800 µL media, briefly vortexed, and diluted 1:2 into 0.4% w/v trypan blue (Sigma-Aldrich, St. Louis MO, USA). The number of viable cells were counted with a hemocytometer.

Free Thiols Assay

Cells were prepared in the same manner as they were for the trypan blue assay. When the media was removed, cells were treated with media containing DMSO, 750 µM H _2_ O _2_ , or 5.29 µM CNP and incubated at 37 °C for 24 hours. As described by Rezaei et al., the treatment media was aspirated, and the cells were washed with cold PBS (2022). Then, 100 μL ice-cold lysis buffer (50 mM Na _2_ HPO4•7H _2_ O (Flinn Scientific, Batavia IL, USA), 1 mM EDTA (ThermoFisher), pH 6.5, 0.1% v/v Triton X-100 (Sigma-Aldrich)) was added to each well. The wells were scraped, and the lysates were collected. Each lysate was incubated on ice for 15 minutes and centrifuged at 10,000 x g for 15 minutes at 4 °C. Lysates were stored at -80 °C for 24 hours. In a 96-well plate, 50 μL of duplicate lysates were mixed with 40 μL of reaction buffer (0.1 M Na _2_ HPO4•7H _2_ O and 1 mM EDTA, pH 8) and 10 μL of 4 mg/mL Ellman’s reagent (5,5′-dithio-bis-(2-nitrobenzoic acid), MedChemExpress) in reaction buffer, including 3 blank wells. The plate was incubated for 15 minutes at 37 °C. Absorbance was measured at 412 nm and normalized to control wells.

*In Silico* Docking Simulation

CB-Dock2 was used to model interactions between CNP (ZINC ID: ZINC000004098049) and NF-κB (PDB: 1SVC). The predicted structure was downloaded from CB-Dock2 (Liu et al., 2022; Yang et al., 2022b) and visualized in ChimeraX (Meng et al., 2023).

Data Analysis

All statistical analyses were performed in GraphPad Prism version 11.0.0. Normality in the data was tested for using the Shapiro-Wilk test, and significance between multiple groups was determined by one-way ANOVA followed by Dunnett’s multiple comparison tests where * represents p < 0.001 and # represents p < 0.0001. CNP concentrations that are not denoted were not significantly different from the control group (p ≥ 0.05). Significance between two groups was determined by a two-tailed, unpaired Student's t-test, and *** represents p < 0.001. Error bars represent standard deviation. All experiments were performed in three independent biological replicates with at least three technical replicates.
